# Vaccine access shrinks disparities between long-term care and community rates of COVID-19 mortality.

**DOI:** 10.1093/geroni/igab046.3651

**Published:** 2021-12-17

**Authors:** Nicholas Resciniti, Daniel Kaplan, Joshua Sellner, Matthew Lohman

**Affiliations:** 1 University of Southern California, Los Angeles, California, United States; 2 Adelphi University, Cortlandt Manor, New York, United States; 3 Arnold School of Public Health, University of South Carolina, Columbia, South Carolina, United States; 4 University of South Carolina, Columbia, South Carolina, United States

## Abstract

This longitudinal secondary data analysis examines differences in COVID-19 incidence and mortality among long-term care facility (LTCF) residents with those living in the community in South Carolina (SC) throughout the pandemic, including the time of vaccine availability. Data came from the SC Department of Health and Environmental Control (SCDHEC). Descriptive statistics and trends for cases of infections and deaths were calculated. Cox proportional hazards were used to compare COVID-19 mortality in LTC residents to community dwelling older adults, controlling for age, gender, race, and pre-existing chronic health conditions. Until early January of 2021, significantly greater incidence rates of infection (116.2 per 10,000 per month) and hazard of death after infection (HR=1.83, 95% CI: 1.70-1.98) were experienced among LTC residents as compared to older adults in the community even after statewide mask mandates and visitation guidance. Since vaccine availability, COVID incidence rates among LTC residents fell by half (59.5 per 10,000 per month after vaccines), and the relative hazard of death compared to older adults in the community was diminished (HR=1.44, 95% CI:1.29-1.61). Reducing the gap between LTCF and community-wide infection and mortality rates suggests that vaccination against COVID-19 is correlated with reduced disease spread in the greater community and in LTCF. Results indicate that policies and regulations addressing LTC resident and staff vaccination may effectively protect the most vulnerable older adults and the workforce providing their care while mask mandates and visitation guidance do not.

